# Behavioral assessment of neuropsychiatric outcomes in rodent stroke models

**DOI:** 10.1177/0271678X251317369

**Published:** 2025-03-20

**Authors:** Robert M Callaghan, Huiyuan Yang, Rachel D Moloney, Christian Waeber

**Affiliations:** 1 School of Pharmacy, 8795 University College Cork, Cork, Ireland; 2 Department of Pharmacology and Therapeutics, School of Medicine, University College Cork, Cork, Ireland; 3 APC Microbiome Ireland, University College Cork, Cork, Ireland

**Keywords:** Anxiety-like behaviour, cognitive behaviour, depressive-like behaviour, post-stroke anxiety, post-stroke depression

## Abstract

Stroke-associated mood disorders are less recognised than sensorimotor impairment, despite their high prevalence. Similarly, few experimental stroke studies assess non-sensorimotor functions. This study examined the prevalence and implementation of non-sensorimotor tests in three stroke-focused journals over the last twenty years. Of 965 experimental ischaemic stroke papers which used behavioural testing in rodents, 932 included sensorimotor testing, while 137 used non-sensorimotor tests (most commonly the Morris water maze, open field, Y-maze, and novel object recognition tests, but with a more diverse range of tests introduced in recent years). Cognition, anxiety and depression were assessed in 70%, 27% and 3% of these 137 papers. Non-sensorimotor deficits were typically observed after recovery of sensorimotor function. Potential confounding factors and challenges for data interpretation were identified in the most prevalent tests. More generally, experimental rigor (a priori power calculation, randomisation, blinding, and pre-defined inclusion/exclusion) improved over the years, but remained unsatisfactory with only 26% of studies providing some evidence of adequate statistical power. Furthermore, most studies focused on male animals, limiting external validity. This review confirms the disparity between sensorimotor and non-sensorimotor testing in experimental stroke but shows that the share of the studies including the latter is increasing. It is essential that research into the neuropsychiatric sequalae of stroke addresses methodological issues noted and continues to expand to improve patient outcomes post-stroke.

## Introduction

Stroke is a significant cause of premature death and disability worldwide.^1^ While the sensorimotor consequences of stroke are well-known, stroke survivors often also present with mood disorders. Indeed, approximately 30% of stroke survivors develop post-stroke depression (PSD) and 20% of survivors develop post-stroke anxiety (PSA).^2–4^ PSD and PSA are associated with a reduced quality of life, increased disability and increased risk of suicide.^5–7^ While much progress has been made in the acute management of stroke, comparatively little effort has been dedicated to studying the pathophysiological mechanisms and management of stroke’s non-sensorimotor sequalae, such as PSD or PSA.^8–10^

Despite the high prevalence and impact of PSD and PSA, behavioural assessment of recovery following experimental stroke seldom models its neuropsychiatric consequences, with most research focusing on alleviating sensorimotor deficits. Sensorimotor complications affect up to 80% of survivors,^11^ warranting a focus on such consequences, but patients suffering from neuropsychiatric conditions post-stroke have so far derived little benefit from experimental stroke research. Incorporating non-sensorimotor testing could offer novel insights into these under-researched conditions while possibly further improving sensorimotor recovery, as patients suffering from PSD/PSA are less likely to engage in rehabilitation programmes if PSD/PSA is left untreated.^6^

Poor experimental quality and rigour have been highlighted as a major reason behind the poor translatability of preclinical stroke research as a whole.^12,13^ In an effort to address these issues, guidelines have been released in recent years including the Stroke Recovery and Rehabilitation Roundtable (SRRR) translational working group guidance, and Stroke Therapy Academic Industry Roundtable (STAIR) preclinical recommendations.^14,15^ These parallel a more general effort to improve reproducibility and translation of animal research, such as the PREPARE and the ARRIVE guidelines.^16,17^

In contrast to the establishment of guidance for sensorimotor testing, there are currently no guidelines focusing on non-sensorimotor testing.^14^ To determine whether there is a need for such guidelines, and possibly address this disparity, we must first understand the extent to which experimental stroke papers assess non-sensorimotor testing. Additionally, assessing the quality of the non-sensorimotor tests currently being used could inform the development of future guidelines.

The purpose of this systematised review was to assess the prevalence of non-sensorimotor testing over the last twenty years (2003–2023 inclusive) in three peer-reviewed stroke-focused journals, to test the hypothesis that behavioural non-sensorimotor testing is less prevalent than sensorimotor assessment. In addition, we aimed to assess the quality and rigour of non-sensorimotor testing, and possibly identify potential confounding factors associated with these tests as a first step towards the development of specific guidelines into non-sensorimotor testing in preclinical stroke models.

A systematised review was chosen due to the exploratory nature of this project, which precluded the implementation of a fully systematic review.^18^ However, our systematised review incorporates many aspects of a fully systematic review, including a broad scope of the literature, a priori defined search strategy, and explicit inclusion/exclusion criteria, increasing the rigour of this review.

Ultimately, improving the implementation of non-sensorimotor behavioural tests in experimental stroke studies should contribute to improving patient outcomes by not only focusing on sensorimotor complications, but also on the common but understudied neuropsychiatric consequences of stroke.

## Materials and methods

### Screening method

**Figure 1.**
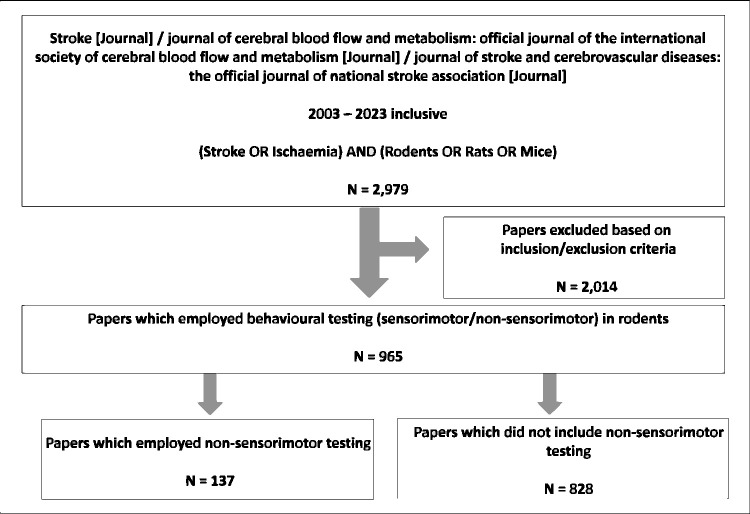
Systematised review methodology. The search strategy implemented in PubMed for peer-reviewed experimental stroke research articles published in three journals (Stroke, Journal of Cerebral Blood Flow and Metabolism, Journal of Stroke and Cerebrovascular Diseases) (2003 – 2023 inclusive) yielded 2,979 papers. Application of inclusion/exclusion criteria eliminated 2,014 papers, leaving 965 original research papers which reported the use of behavioural tests in rodents following experimental stroke. Of these papers 137 reported the use of non-sensorimotor behavioural testing; they were subsequently selected for more extensive analysis as described.

The scope of this systematised review was limited to three journals focused on stroke, namely Stroke (published by Lippincott Williams & Wilkins), the Journal of Cerebral Blow Flow and Metabolism (published by Sage), and the Journal of Stroke and Cerebrovascular Diseases (published by Elsevier). PubMed® (NIH National Library of Medicine) was used to search for relevant papers using the search strategy ((Stroke OR Ischaemia) AND (Rodents OR Rats OR Mice) AND (“Journal of cerebral blood flow and metabolism: official journal of the International Society of Cerebral Blood Flow and Metabolism”[Journal] OR “Stroke”[Journal] OR “Journal of stroke and cerebrovascular diseases: the official journal of national stroke association”)). This search was limited to papers published between 2003–2023, yielding a total of 2,979 papers which were individually screened based on the below inclusion/exclusion criteria (Figure 1).

### Inclusion/exclusion criteria

Inclusion criteria were: use of a surgical method to produce brain ischaemia in rats or mice, and the use of behavioural testing (either sensorimotor/non-sensorimotor testing) following the occlusion. Exclusion criteria were: review papers, paper duplicates, and research which investigated cerebrovascular diseases distinct from ischaemic stroke including haemorrhagic stroke, hypoxia-ischaemia, vascular dementia, cardiac arrest and Alzheimer’s disease. Finally, papers which included rats/mice but focused solely on lesion size (histology/in vivo imaging) without performing behavioural testing were also excluded. Following the application of these inclusion/exclusion criteria, 965 papers were selected for full-text analysis.

### Data management

Data was extracted from eligible papers and compiled into a Microsoft Excel spreadsheet. Of the 965 papers chosen for full-text analysis, 828 did not employ non-sensorimotor behavioural testing. The only data extracted from these papers were the year of publication and the sensorimotor tests employed. The data extracted from the remaining 137 papers, which included non-sensorimotor behavioural testing, included year of publication, details regarding the occlusion method employed, duration of occlusion, and the side of infarction. Data was also collected pertaining to the species, strain, age, and sex of rodents used for experimentation. The sensorimotor and non-sensorimotor tests employed and what period after stroke these tests were employed were also recorded. In order to determine whether sensorimotor and non-sensorimotor deficits temporally overlap, 4 time points were collected: first day of behavioural testing, the first and the last day a significant difference was observed, and the last day of behavioural testing (all expressed as days post-occlusion; tests that did not specify post-stroke timing were not included in the time analysis). Additional data specific to the most prevalent behavioural tests was also collected, e.g., the apparatus and rodent training protocol employed for the Morris Water Maze, the level of lighting and behaviours measured for the open field test, and the nature of the novel object used within the novel object recognition test. Finally, information regarding general experimental rigour was collected, including specific information on the randomisation method, blinding, inclusion/exclusion criteria, and evidence of a priori group size (power) calculations. Data not reported in the main article or its supplemental material (when available) was denoted “not specified”.

### Data and statistical analysis

Raw data following screening was processed in Excel to quantify how often individual non-sensorimotor tests were used, the stroke model employed, species, sex, strain, and rodent age, as well as data pertaining to general experimental rigour as described above. Once quantified this data was transferred to GraphPad Prism (GraphPad Software, Version 10.2.3) and graphed. Venn Diagram plotter was used to produce a quantitative Venn Diagram to categorise papers assessed for full-text analysis. The experimental timeline of behavioural tests was analysed using SPSS (IBM, Version 28). Descriptive statistics were then used to determine the median value and interquartile ranges for the four mentioned timepoints post-stroke. The median value was chosen as the timeline data was not normally distributed as determined by the Shapiro-Wilk and Kolmogorov-Smirnov tests for normality (p < 0.001). In one case the behavioural tests conducted at different timepoints in male and female animals; these were analysed separately. The Mann Whitney U test was used to compare the distributions of sensorimotor versus non-sensorimotor tests during the four time points. Experimental timelines were then displayed using cluster box plots with interquartile ranges and individual data points using GraphPad Prism.

## Results

### Model of occlusion employed, and brain hemisphere affected

**Figure 2.**
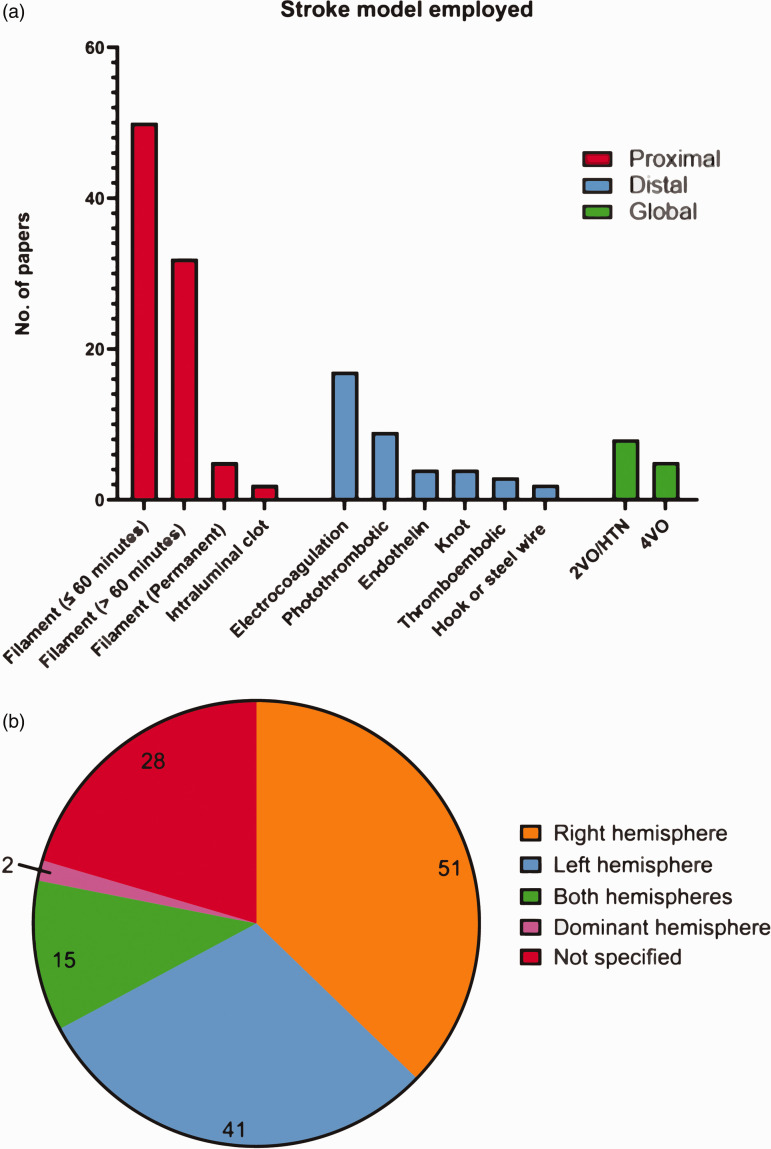
Prevalence of occlusion methods used to mimic stroke in rodents. (a) The number of papers employing different stroke models (N = 141; two papers employed two monofilament occlusion times, one paper employed both a distal thromboembolic and electrocoagulation model, and one paper employed both a monofilament and electrocoagulation model) and (b) Hemisphere targeted for infarction (N = 137 papers which included non-sensorimotor behavioural testing). Abbreviations: 2-vessel occlusion with hypertension (2VO/HTN), 4-vessel occlusion (4VO).

We found 137 papers which included non-sensorimotor behavioural testing; of these, 87 (64%) employed proximal models of ischaemia (filament occlusion, intraluminal clot), 38 (28%) used distal models (electrocoagulation, photothrombotic, endothelin, knot, thromboembolic, hook or steel wire occlusion), and 13 (9%) used global models (2-vessel occlusion with hypotension, 4-vessel occlusion) (Figure 2(a)). One paper employed both a proximal and distal model. The filament occlusion method was the most employed (85 papers, 62%). This was followed by the electrocoagulation and photothrombotic occlusion, with 17 (12%), and 9 (7%) papers respectively. Less common stroke models included 2-vessel occlusion with hypotension (8 papers, 6%), 4-vessel occlusion (5 papers, 4%), topical endothelin (4 papers, 3%), knot (4 papers, 3%), thromboembolic (3 papers, 2%), intraluminal clot (2 papers, 1%), and hook or steel wire (2 papers, 1%).

For the filament method, we clustered the data into three distinct groups, to account for the increasing extent of the damage: 1) ≤60 minutes occlusion whereby infarction is generally smaller and primarily affects the striatal area (50 papers), 2) >60 minutes occlusion whereby infarctions are larger, generally affecting both the striatum and the cortex (32 papers), and 3) permanent occlusion in 5 papers (being cognisant that experimental conditions and the experimenter can also affect the relationship between ischemia duration and infarct size/location). One paper did not indicate ischemia duration, which was clarified by contacting its authors (Figure 2(a)).

The right hemisphere was most commonly infarcted, accounting for 51 (37%) papers, with the left hemisphere being infarcted in 41 (30%). Both hemispheres were affected simultaneously in 15 (11%) papers, while 2 (1%) papers targeted the hemisphere of the brain contralateral to a rodent’s dominant front paw, as determined by a pre-stroke assessment. A large portion of papers (28, or 20%) did not specify the hemisphere targeted for infarction (Figure 2(b)).

### Prevalence of non-sensorimotor behavioural testing in the stroke literature, and temporal patterns

**Figure 3.**
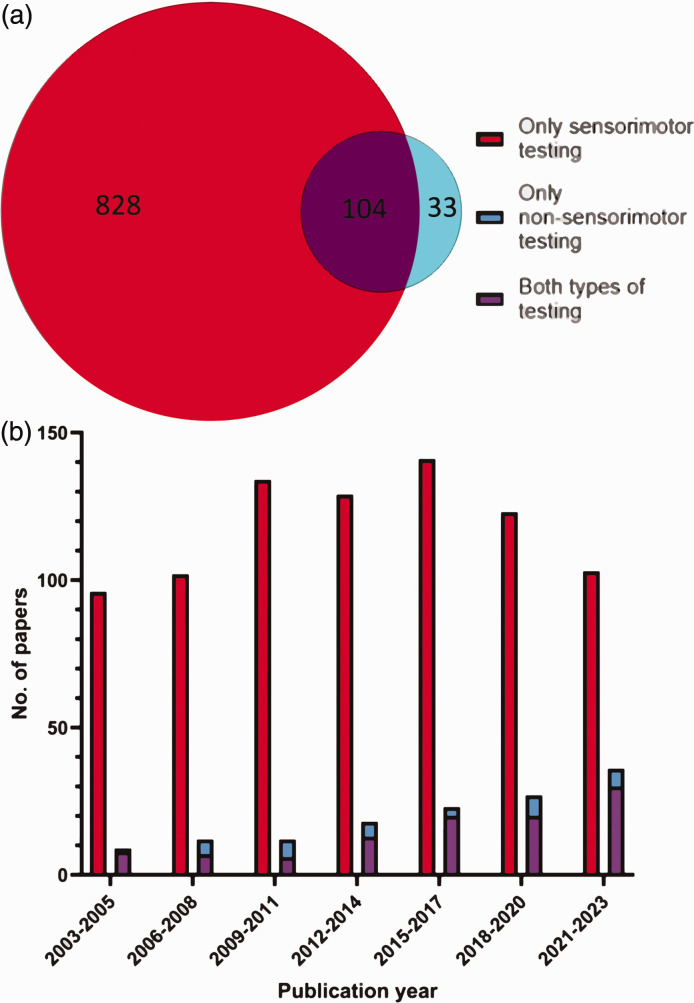
Prevalence of sensorimotor and non-sensorimotor testing. (a) Number of papers selected for full-text analysis over the 20-year period that employed sensorimotor and/or non-sensorimotor testing (N = 965) and (b) Number of papers in a given three-year period employing sensorimotor, non-sensorimotor and both types of behavioural testing respectively. **2003–2005** (96 papers employing sensorimotor testing only, 1 paper employing non-sensorimotor testing only, 8 papers employing both sensorimotor and non-sensorimotor testing), **2006–2008** (102 papers employing sensorimotor testing only, 5 papers employing non-sensorimotor testing only, 7 papers employing both sensorimotor and non-sensorimotor testing), **2009–2011** (134 papers employing sensorimotor testing only, 6 papers employing non-sensorimotor testing only, 6 papers employing both sensorimotor and non-sensorimotor testing), **2012–2014** (129 papers employing sensorimotor testing only, 5 papers employing non-sensorimotor testing only, 13 papers employing both sensorimotor and non-sensorimotor testing), **2015–2017** (141 papers employing sensorimotor testing only, 3 papers employing non-sensorimotor testing only, 20 papers employing both sensorimotor and non-sensorimotor testing), **2018–2020** (123 papers employing sensorimotor testing only, 7 papers employing non-sensorimotor testing only, 20 papers employing both sensorimotor and non-sensorimotor testing), and **2021–2023** (103 papers employing sensorimotor testing only, 6 papers employing non-sensorimotor testing only, 30 papers employing both sensorimotor and non-sensorimotor testing).

Of 2,979 papers screened, 965 (32%) reported behavioural testing on rodents following the induction of ischaemic stroke; 932 (97%) of these included sensorimotor testing, while 137 (14%) included non-sensorimotor testing. Breaking these down, 828 (86%) papers employed only sensorimotor testing, 33 (3%) papers used non-sensorimotor testing only, and 104 (11%) papers employed both sensorimotor and non-sensorimotor tests (Figure 3(a)). Of the 965 papers reporting behavioural testing over the past 20 years the number of papers using non-sensorimotor testing steadily increased over the 20-year study period, growing from 9 (2003–2005) to 36 (2021–2023) over the respective 3-year periods. (Figure 3(b)).

A total of 195 non-sensorimotor behavioural tests were conducted in the 137 papers reporting the use of non-sensorimotor tests. The Morris water maze was the most prevalent non-sensorimotor test, reported in 65 (47%) papers. This was followed by the open field test (48 (35%)), Y-maze (19 (14%)), novel object recognition test (16 (12%)), and passive-avoidance test (10 (7%)). Less common non-sensorimotor tests included: Barnes maze (7, (4%)), fear conditioning test (8, (4%)), elevated plus maze (5, (3%)), t-maze (4, (2%)), novel odour recognition test (2, (1%)), touchscreen test (2, (1%)), sucrose preference test (2, (1%)), forced swim test (2, (1%)), radial arms maze (1, (0.5%)), Lashley III maze (1, (0.5%)), three chamber test (1, (0.5%)), novel location recognition (1, (0.5%)), and tail suspension test (1, (0.5%)). Descriptions, references, and depictions of these non-sensorimotor tests are provided in Table 1 of the Supplementary Material; readers interested should also refer to other reviews.^19,20^

**Figure 4.**
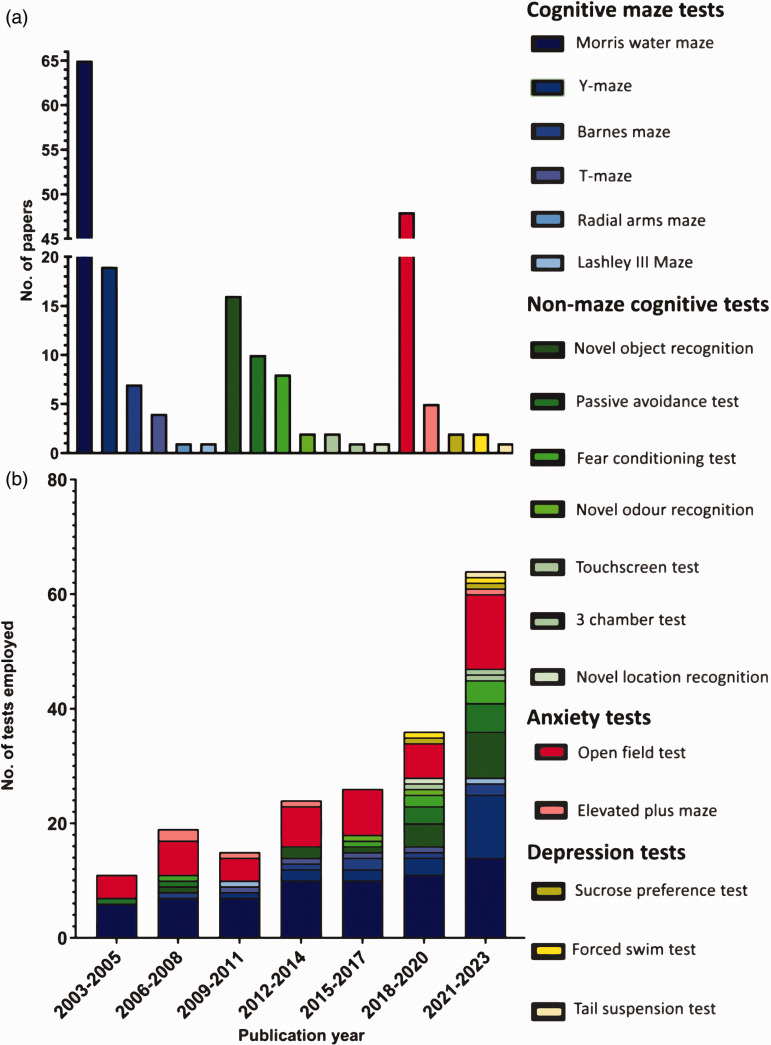
Prevalence of individual non-sensorimotor tests. (a) Number of times individual non-sensorimotor tests were employed throughout the assessed period (2003–2023 inclusive) and (b) total number of times individual tests were employed in all included papers published during a given 3-year period (N = 195). Cognitive maze tests in shades of blue, non-maze cognitive tests in shades of green, anxiety tests in red, depression tests in yellow. **2003–2005** (Morris water maze: 6, passive avoidance test: 1, open field: 4). **2006–2008** (Morris water maze: 7, Barnes maze: 1, novel object recognition: 1, passive avoidance test: 1, fear conditioning test: 1, open field: 6, elevated plus maze: 2). **2009–2011** (Morris water maze: 7, Y-maze: 1, t-maze: 1, radial arms maze: 1, open field: 4, elevated plus maze: 1). **2012–2014** (Morris water maze: 10, y-maze: 2, Barnes maze: 1, t-maze: 1, novel object recognition: 2, open field: 7, elevated plus maze: 1). **2015–2017** (Morris water maze: 10, y-maze: 2, Barnes maze: 2, t-maze: 1, novel object recognition: 1, fear conditioning test: 1, novel odour recognition: 1, open field: 8). **2018–2020** (Morris water maze: 11, y-maze: 3, Barnes maze: 1, t-maze: 1, novel object recognition: 4, passive avoidance: 3, fear conditioning: 2, novel odour recognition: 1, touchscreen: 1, novel location recognition: 1, open field: 6, sucrose preference: 1, forced swim test: 1). **2021–2023** (Morris water maze: 14, y-maze: 11, Barnes maze: 2, Lashley III maze: 1, novel object recognition: 8, passive avoidance: 5, fear conditioning: 4, touchscreen: 1, three chamber: 1, open field: 13, elevated plus maze: 1, sucrose preference: 1, forced swim: 1, tail suspension: 1).

Tests predictive of cognitive impairment were the most commonly used, with 70% (137) of all non-sensorimotor tests assessing cognitive behaviours using an array of tests. Of these, 97 tests were maze-based (shades of blue in Figure 4), primarily assessing spatial learning and memory (the Morris water maze, Y-maze, Barnes maze, T-maze, radial arms maze, and Lashley III maze) while 40 used other behavioural tests to assess other aspects of cognition and memory (shades of green) (novel object recognition, fear conditioning test, passive avoidance test, novel odour recognition, and operant touchscreen test). Tests assessing anxiety (shown in red) (open field test, and elevated-plus maze) accounted for 27% (53), while tests assessing depressive-like behaviours (shown in yellow) (sucrose preference test, forced swim test, and tail suspension test) accounted for 3% (5) of the total 195 non-sensorimotor tests employed (Figure 4(a)).

While the Morris water maze and open field test have remained widely used throughout the last 20-year period, the variety of non-sensorimotor tests being reported has increased, from 3 different behavioural tests in all papers published between 2003 and 2005 to 14 different non-sensorimotor tests being employed in the most recent three-year period (2021–2023). The breakdown of the tests used in each three-year period is displayed in Figure 4(b).

Automated software (e.g. AnyMaze and EthoVision XT) was used to analyse 94 (48.2%) behavioural tests, and manual scoring was used for 24 (12.3%) behavioural tests. The method of quantification was not specified in 77 (39.5%) behavioural tests. Behavioural tests that commonly involved automated scoring included the Morris water maze and the open field, novel object recognition, and fear conditioning tests whereas the sucrose preference and forced swim tests were exclusively scored manually (breakdowns of the use of automated vs. manual scoring for each individual test are shown in Table 2 of the Supplemental Material).

### Behavioural assessment timelines

**Figure 5.**
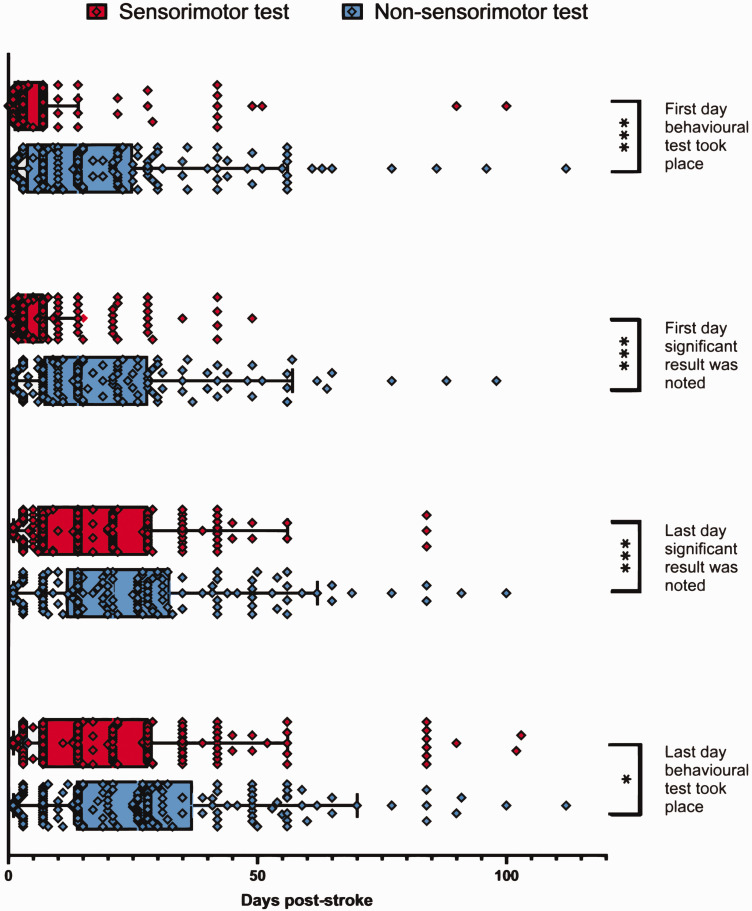
Experimental timelines of both sensorimotor and non-sensorimotor testing. The median and interquartile range (IQR) are shown. Sensorimotor tests employed with collatable results (N = 170); non-sensorimotor tests employed with collatable results (N = 149). Mann Whitney U test was performed comparing sensorimotor and non-sensorimotor distributions for each of the four pre-defined timepoints. *P < 0.05, ***P < 0.001.

Non-sensorimotor behavioural testing tended to be initiated later post-ischaemic induction compared to sensorimotor testing (Figure 5). Papers began sensorimotor testing at a median of 3 days post-stroke (IQR 6) compared to non-sensorimotor tests at a median of 14 days post-stroke (IQR 20) (p < 0.001). Similarly, the first significant effects for sensorimotor tests were reported at an earlier timepoint post-stroke with a median reporting of 3.5 days (IQR 5) compared to 14 days for non-sensorimotor tests (IQR 21) (p < 0.001). The final day a significant effect reported for sensorimotor tests was also at earlier timepoints, with a median of 14 days (IQR 22.25) compared to 21 days post-stroke for non-sensorimotor tests (IQR 21) (p < 0.001). The final day behavioural testing took place post-stroke was earlier for sensorimotor tests at 17 days (IQR 21) compared to non-sensorimotor ones at 26 days post-stroke (IQR 21) (p < 0.05).

### Species, strain, and sex

**Figure 6.**
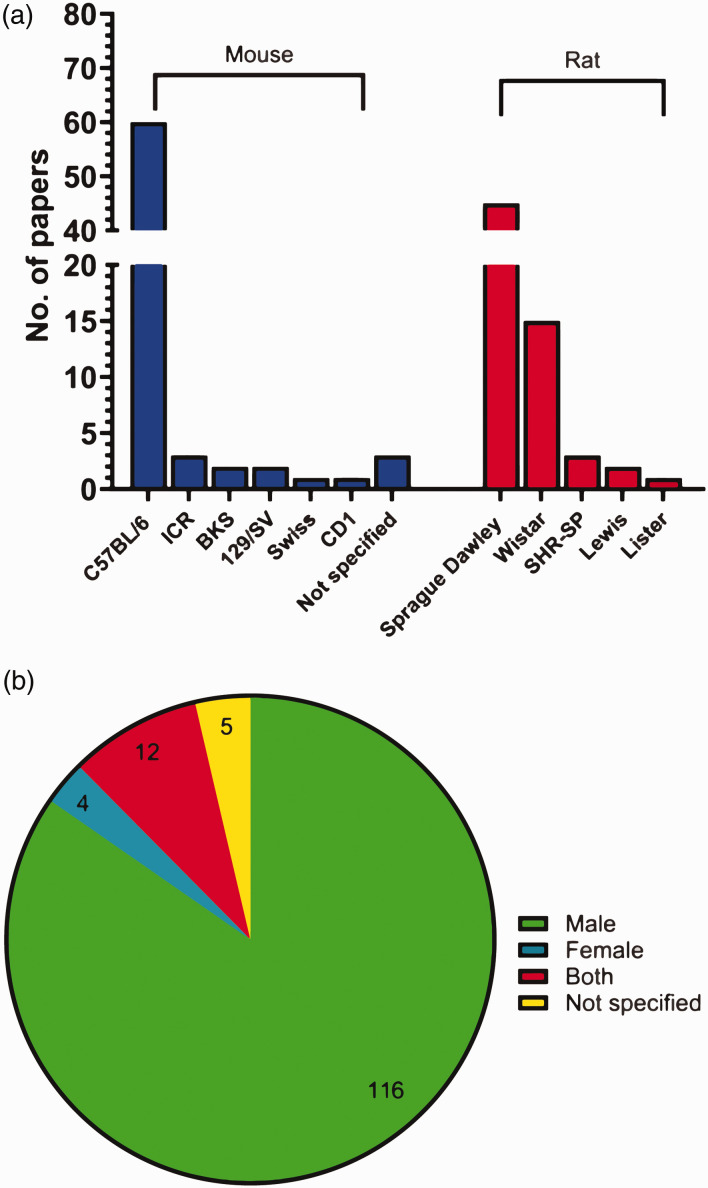
Prevalence of species, strain, and sex of rodent used for non-sensorimotor behavioural testing. (a) Number of papers using each species, and strain of rodent for non-sensorimotor testing (N = 138) and (b) percentage of papers using each sex for non-sensorimotor behavioural testing (N = 137).

Both rodent species were utilised to similar degrees, with 72 (53%) papers using mice and 66 (48%) papers using rats (one paper used both). The C57BL/6 strain (inbred) was the most common mouse strain, used in 60 papers (83% of papers that used mice). Less common mouse strains included: ICR (3 papers), BKS (2 papers), 129/SV (2 papers), Swiss (1 paper), and CD1 (1 paper). 3 papers did not specify the mouse strain used. Sprague Dawley rats (outbred) were the most commonly used rat strain at 68% (45 papers), while less common rat strains included Wistar (15 papers), SHR-SP (3 papers), Lewis (2 papers), and Lister (1 paper) (Figure 6(a)). For C57BL/6 mice, 33 (55%) papers mentioned the sub-strain. Rodents assessed in non-sensorimotor testing were predominantly male, with 116 (85%) papers using only males, 4 (3%) using only females, and 12 (9%) using both males and females (9 of which assessed sex as a factor; 3 papers collapsed males and females into one group without distinguishing sex) (Figure 6(b)).

### General experimental rigour

Conducting rigorous and reproducible preclinical research requires adherence to several minimal requirements including a priori sample size calculation, randomisation, blinding, and pre-defined inclusion/exclusion.^21–23^ We found that 79% (108) of papers reported blinding when conducting experimentation. Randomisation was mentioned in 75% (103) of papers; inclusion/exclusion criteria were described for 66% (91) of papers. These inclusion/exclusion criteria were related to various experimental domains, those relating to the in vivo stroke model itself were the most common, being mentioned in 69 (50%) papers. Criteria related to other aspects of the studies are listed in Table 3 of the Supplemental Material. About one in four papers (32, 26%) indicated that their behavioural tests were adequately powered (loosely defined as either including a specific power calculation, or just specifying that sample sizes were based on previous power calculations used for the same behavioural test).

**Figure 7.**
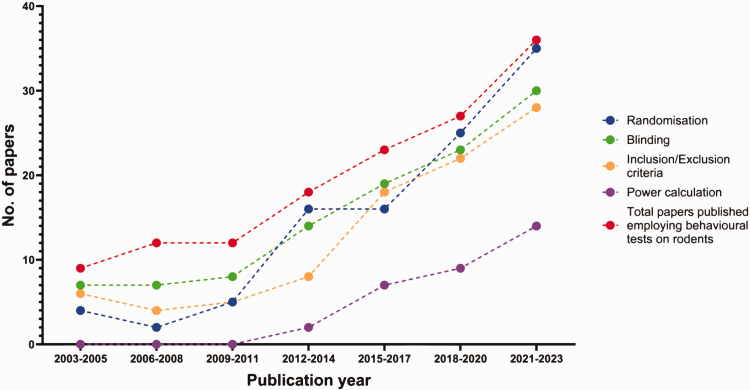
Number of papers mentioning randomisation, blinding, predefined inclusion/exclusion criteria, and power calculations in 3-year periods over the last twenty years (2003–2023) (N = 137). Paper breakdown per three year period **2003–2005** (randomisation 4, blinding 7, inclusion/exclusion criteria 6, power calculation 0, total 9) **2006–2008** (randomisation 2, blinding 7, inclusion/exclusion criteria 4, power calculation 0, total 12) **2009–2011** (randomisation 5, blinding 8, inclusion/exclusion criteria 5, power calculation 0, total 12) **2012–2014** (randomisation 16, blinding 14, inclusion/exclusion criteria 8, power calculation 2, total 18) **2015–2017** (randomisation 16, blinding 19, inclusion/exclusion criteria 18, power calculation 7, total 23) **2018–2020** (randomisation 25, blinding 23, inclusion/exclusion criteria 22, power calculation 9, total 27) **2021–2023** (randomisation 35, blinding 30, inclusion/exclusion criteria 28, power calculation 14, total 36).

Comparing 2003–2005 with 2021–2023 data suggests that experimental rigour has increased in this period. The proportion of papers mentioning blinding increased from 78% to 83%, randomisation increased from 44% to 97%, predefined inclusion/exclusion criteria increased from 66% to 78%, and evidence of appropriately powered experiments increased from 0% to 39% (Figure 7).

### Implementation of the Morris water maze, open field and novel object recognition tests

Given the prevalent use of the Morris water maze, open field, and novel object recognition tests, potential confounding factors and challenges for accurate interpretation of their data were assessed in detail.

For the Morris water maze, important confounding factors in experimental stroke studies include difficulty swimming, thigmotaxis, and visual impairment.^24–26^ Of the 65 papers that employed the Morris water maze, 31 (48%) ruled out a contribution of motor impairment by measuring swim speed, 32 (49%) ruled out thigmotaxis through a swim pattern assessment, and 20 (31%) confirmed visual acuity through the use of a visible platform trial. Only 5 (8%) papers ruled out all three potential confounding factors. While 22 (34%) papers mentioned the use of external spatial cues, only 3 (5%) described the nature of these cues (pictures and lamps, cues with different shapes and colours, and a fixed camera/poster/cabinet/experimenter respectively). The platform was adequately concealed (i.e. a black platform in opaque water, or a transparent platform in non-coloured water) in 28 (43%) papers, while 37 (57%) papers did not specify if the platform was concealed. We also found that 50 (77%) papers trained rodents following induction of an ischaemic stroke, 10 (15%) before induction of ischaemic stroke, 1 (2%) paper trained rodents both before and after ischaemic stroke was induced, and 4 (6%) papers did not specify when training took place. During the training period, only 3 (5%) papers mentioned exclusion of rodents based on failure to learn during the training phase.

**Figure 8.**
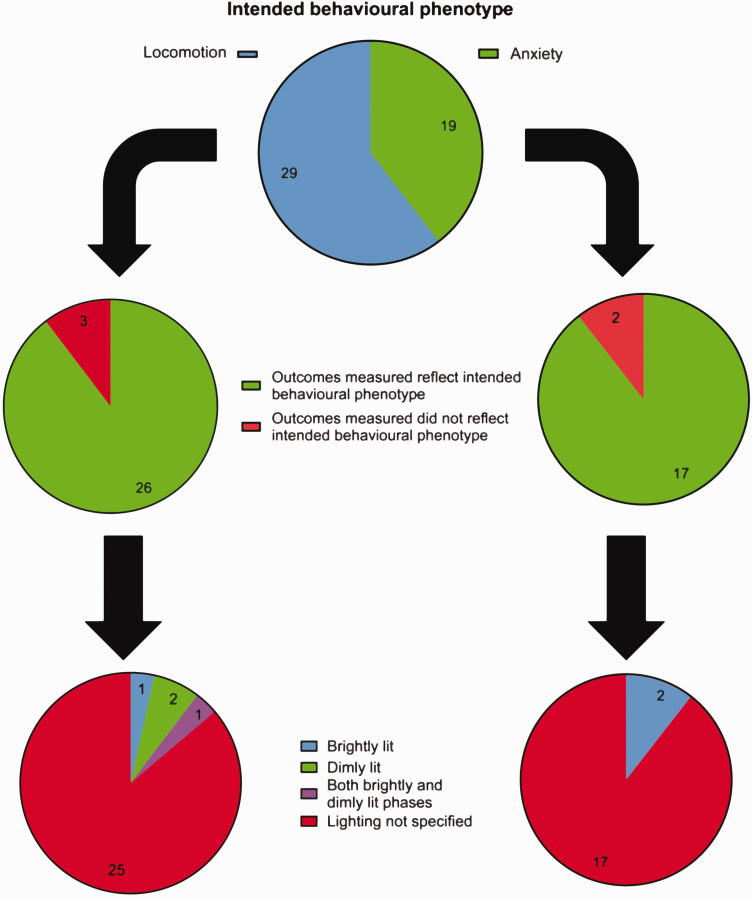
Experimental protocol employed using the open field test. Top) Number of papers using the open field test to assess anxiety levels. Middle) Number of papers showing appropriate outcome measures when intending to assess locomotion (left) and anxiety (right). Bottom) Number of papers specifying lighting levels when intending to assess locomotion (left) and anxiety (right). (N = 49).

The open field test can be used to assess locomotor or anxiety-like behaviour, depending on how it is implemented. Among the papers we analysed, 29 (60%) papers mentioned using it to assess general locomotion without referring to anxiety, while 19 (40%) mentioned using this test to assess anxiety levels. The open field test can yield several parameters, some more directly related to locomotion, others indicative of anxiety-like behaviour.^27^ Most papers used the appropriate parameters to assess the intended behavioural phenotype: 26 (89%) papers assessing locomotion used walking speed, travel distance, number of infrared beam breaks, time spent mobile, and 17 (89%) papers assessing anxiety quantified time spent in the centre of the arena, circling behaviour, rearing, grooming, the number of faecal pellets produced, time spent in the corners of the arena. Lighting intensity in the open field is an essential consideration when assessing anxiety (brightly lit arenas are typically used to assess anxiety-like behaviours, while dimly or red-lit arenas are more appropriate when assessing general sensorimotor functioning).^28–30^ However, lighting levels were rarely specified, with only 6 (12%) reporting intensity. Of those papers which assessed anxiety only 2 specified the appropriate lighting level (brightly lit), while only 2 papers intending to assess locomotion specified the correct lighting (dimly lit) (Figure 8).

Various parameters of the novel object recognition test often vary between papers, and these have been suggested to impact on results.^31^ Five (31%) papers specified the nature of the novel object introduced. When assessing the time between the exploration test (i.e. two identical objects in the arena) with the novel object recognition test (i.e., one of the items replaced with a new novel object) we found that 2 (12.5%) papers separated these two tests by minutes (five minutes, and fifteen minutes respectively), 6 (37.5%) papers separated them by hours (one hour in 5 papers and 2 hours in one paper), and 7 (43.75%) papers allowed one day between the test phases (with one paper also conducting the test again at 7 days post exploration phase). One (6.25%) paper did not specify how much time separated the exploratory and novel object phases. The exploration time given to rodents varied: 2 (12.5%) papers allowed three minutes to explore, 9 (56.25%) allowed five minutes, 1 (6.25%) allowed eight minutes, and 2 (12.5%) allowed ten minutes to explore the arena with the novel object. Two (12.5%) papers did not specify this duration. Few papers defined what constituted novel object exploration with two (12.5%) papers measuring time oriented toward the novel object, one (6.25%) measuring time touching or orienting towards the novel object, one (6.25%) measuring time sniffing and orienting towards the novel object, one (6.25%) measuring time on the novel object, while 11 (68.75%) measured time spent investigating the novel object without defining what constituted exploration. Although an overhead camera angle is essential for optimal view of exploration, it was rarely specified with 4 (25%) papers specifying an overhead angle, while 12 (75%) did not specify camera angle.^32^

## Discussion

The goal of this review was to test the hypothesis that translational research into the neuropsychiatric outcomes post-stroke is less prevalent than studies of its sensorimotor consequences. To do so, we assessed the prevalence of non-sensorimotor testing over the last 20 years (2003–2023 inclusive) in three peer-reviewed stroke-focused journals. Assessment of the neuropsychiatric consequences of stroke only accounted for 14% of all papers selected for full text analysis (i.e., reporting the use of behavioural testing), but this prevalence has been increasing in recent years. The most popular non-sensorimotor tests were the Morris Water maze, the open field test, Y-maze, and the novel object recognition test. While the Morris water maze and open field test have remained by far the most commonly used tests over the study period, the Y-maze and novel object recognition test have grown in popularity in recent years.

Behavioural scoring methods varied across non-sensorimotor tests. Many papers failed to specify whether scoring was done through automated software or manually, although details on the scoring approach employed would improve transparency and facilitate reproducible research into neuropsychiatric outcomes post-stroke. Of those that did specify, automation was commonly used. While automatic software can allow for efficiency and a reduction of human bias, its cost and limited sensitivity to detect subtle behaviours must be kept in mind. For example, tests like the forced swim test involve a behavioural shift from swimming to freezing that is difficult to detect with current automated software.^33^ Additionally, environmental factors such as lighting, and contrast with background can significantly impact the quality of automated tracking.^34,35^ However, recent advances have improved automated tracking, with deep learning enabling experimenters to label body parts of interest, making “markerless” tracking behaviours such as social interactions, grooming, and climbing possible.^36–38^

Different types of non-sensorimotor sequelae of stroke (cognitive impairments, anxiety, and depression) were not being addressed to similar degrees. Cognitive functioning was assessed in most non-sensorimotor tests employed (70%), followed by anxiety (27%). In comparison, our review found that only 3% of non-sensorimotor tests assessed post-stroke depression like behaviours. This is surprising in light of the high prevalence of post-stroke depression (PSD), which occurs in 30–50% of survivors.^4,39^ While it is hard to say why post-stroke depression is rarely assessed in preclinical research, the relative lack of behavioural tests available to measure a depressive phenotype may be a factor. While cognition and anxiety can be measured by a plethora of straightforward tests with clear readouts, the forced swim, tail suspension and sucrose preference tests are the most commonly used to assess depressive-like phenotype or predict efficacy of potential antidepressant interventions.^40–42^ The suitability of the forced swim and tail suspension tests for these purposes is also being questioned.^43^ In the forced swim test, it may also be difficult to consistently score immobility, resulting in possible observer bias. Other factors such as testing duration, scoring duration, water temperature, and handling conditions can also affect results.^44^ The tail suspension test is only suitable for mice (rats are too heavy), and the sucrose preference test only provides a measure of anhedonia (which is only one of the facets of PSD).

The clinical features of depression can vary from patient to patient; similarly, PSD may present with alterations in motivation, energy, sleep patterns, and/or lead to increased despair and suicidality, and should probably not be treated as a single entity.^45,46^ Thus the field of experimental neuropsychiatry has moved to a more holistic approach implementing multiple tests to assess the multi-facetted aspects depression, with the sucrose preference test modelling anhedonia, “in/escapable shock” modelling learned helplessness, and newly developed tests such as the sinking platform test to model resilience to stress.^42,47–51^ Other tests that could be considered include the nest building test as a measure of motivation and self-care, and sleep patterns via EEG technology.^52,53^ Continuous home cage monitoring systems offer an approach less sensitive to environmental confounders when compared to traditional behavioural tests, and have the ability to identify behaviours specific to phases of the menstrual and circadian cycles.^54^ Operant wall systems can assess working memory, flexibility, time-keeping, and feeding behaviour while computerised visual systems have allowed assessment of locomotion, avoidance behaviour, grooming, and sleep patterns.^55–58^ The use of radiofrequency identification tags has also allowed assessment of memory, impulsivity, and addiction-related behaviours in group housed rodents (a significant advancement as single housing was traditionally required for homecage monitoring systems).^59–63^ Given PSDs high prevalence and its effect on quality of life and mortality rates of stroke survivors, the implementation of these behavioural tests or the development of novel ones might enable the researcher to model the various aspects of PSD.^64–66^

While assessing the neuropsychiatric consequences of experimental stroke is becoming more common, studies of sensorimotor deficits still predominate. The increase in non-sensorimotor testing was largely accounted for by papers employing both sensorimotor and non-sensorimotor tests. We also found an increase in the diversity of non-sensorimotor tests used in recent years, going from three tests (2003–2005) to 14 different non-sensorimotor tests in the most recent three-year period (2021–2023). While this may be beneficial to mimic the complexity of the clinical situation, as mentioned above, using batteries of tests could increase the likelihood of Type I errors.^67^ This risk is compounded by the fact that many tests yield a multiplicity of readouts that may not always be selected before starting the study. Multiple behavioural testing and extensive animal handling could also increase animal stress and anxiety, which might confound the results of the tests aimed at quantifying the behavioural consequences of stroke.^68,69^

An increasing variety of tests also adds to the challenge of comparing across studies. Even when the same behavioural test is used, results can vary across laboratory. In the context of pain research, for example, variables such as housing, diet, pre/postnatal stress, circadian rhythm and sex of the experimenter have been shown to confound the data.^70^ At the very least, environmental stressors should be minimised or at least accounted for when studying PSD and PSA in rodents, since stress has been shown to play a role in these conditions.^71–73^ Another consideration in stroke is the potential confounder of motor dysfunction,^74,75^ which can affect performance in tests such as the Morris water maze.^25^ Yet, we found that many studies did not show evidence of controlling for confounders such as difficulty swimming, thigmotaxis, and visual impairment in the Morris water maze. While the recent recommendations of Stroke Recovery and Rehabilitation Roundtable provide guidance on preclinical sensorimotor testing in stroke models, similar guidelines on non-sensorimotor testing would be a useful resource to address some the issues highlighted above.^14^

This study found that sensorimotor testing typically begins earlier post-stroke compared to non-sensorimotor assessment, with a large portion of papers conducting non-sensorimotor tests after the first week post-stroke. Sensorimotor deficit often recovers within a couple of weeks post-stroke; this recovery could be harnessed to decrease the likelihood sensorimotor dysfunction confounds neuropsychiatric testing.^76–78^ The final day significant differences were reported occurred later for non-sensorimotor than for sensorimotor tests, suggesting that stroke can cause genuine neuropsychiatric deficits independent of sensorimotor ones. Our findings also mimic the clinical situation, whereby the neuropsychiatric sequalae of stroke present months post-stroke while sensorimotor impairment is observed earlier.^79–82^

To optimise non-sensorimotor tests several measures can be put into place to minimise the confounding effects of sensorimotor impairment. Based on our results it would be prudent to wait a few weeks for sensorimotor recovery before beginning neuropsychiatric testing.^77,78^ Behavioural tests less reliant on locomotor function (nest building, sucrose preference, novel object recognition, and homecage monitoring) might be preferable to those that are (Morris water maze, and forced swim test).^83^ If multiple behavioural tests are used, more “active” tests like the aforementioned Morris water maze should be performed last-allowing additional time for motor function to recover. Additionally, sensorimotor assessment should be conducted prior to non-sensorimotor testing to confirm the absence of impairment.^84^ The stroke model employed should be considered, with proximal/global models typically leading to extensive striatal damage, and more pronounced sensorimotor impairment compared to more distal models.^85–90^

The rodent sub-strain was often not reported, despite the fact that differences in behavioural phenotypes have been observed between sub-strains across many neuropsychiatric outcomes such as anxiety, depressive-like behaviour, and spatial working memory.^91^ Likewise, the majority of papers included only male rodents, despite women (particularly post-menopausal) being disproportionally affected by stroke and its sequalae.^92–94^ Of the few papers analysed that included females, only three accounted for the oestrous cycle, which may affect performance in non-sensorimotor testing. For example, C57BL/6 mice show more depressive behaviour during the metestrus phase when using the tail suspension test, while cognitive performance is reduced in the oestrous phase.^95^ Possible experimental variability due to the oestrous cycle should however not be used as a reason to use only males in animal models.^96,97^ While ovariectomy could be used to study the effects the menopause has on stroke outcomes, it is commonly utilised in young rodents-which does not replicate the gradual hormonal decline seen in post-menopausal women.^98^ Instead, aged rodents should be used to more accurately mimic the menopause in humans, making findings more translationally relevant.^99^

We also noted issues related to overall experimental rigour. Most recent papers had controls in place such as blinding, randomisation, and the definition of inclusion/exclusion criteria. However, inclusion/exclusion criteria were inconsistent with many papers only implementing criteria on surgical outcome. More comprehensive, a priori defined, inclusion/exclusion criteria outcomes would increase study reproducibility, and enhance experimental rigour. Few papers provided evidence that tests were adequately powered. Underpowering remains an issue, with a median statistical power of neuroscience studies between 8 and 31%.^23^ This can lead to an overestimated effect size, decreased reproducibility, and also has ethical implications. Future experimental stroke studies should therefore provide detailed power calculations, include both male and female animals, while also reporting details such as the specific sub-strains used, in line with STAIR and ARRIVE recommendations.^15,17^

While this systematised review highlights a research gap in the experimental stroke field by showing the disparity between sensorimotor and non-sensorimotor testing, it has several limitations. One potential limitation is the limited scope of journals included. Journals more largely focused on behavioural neuroscience were not included due to a lack of resources. A fully systematic review could potentially have drawn different conclusions on the disparity between sensorimotor and non-sensorimotor testing in experimental stroke, the range of tests employed and/or attention to potential confounders.

One in four people are now estimated to have a stroke in their lifetime. Of those that survive, 30–50% will present with PSD, and approximately 20% will develop symptoms of PSA. While therapeutic interventions that reduce stroke severity and the sensorimotor consequences of stroke may reduce the incidence of PSD and PSA, as has been shown with alteplase, non-sensorimotor sequalae might benefit from specific interventions.^100,101^ These will more likely be developed based on a better understanding of their pathophysiology, and the use of robust and relevant non-sensorimotor tests. While the STAIR and SRRR guidelines have enhanced the clinical relevance of preclinical stroke research by fostering the inclusion of behavioural testing, they have largely been associated with research into the sensorimotor impairments of stroke. Despite its heavy burden on stroke survivors, research into the non-sensorimotor sequalae of stroke remains much less prevalent. More efforts, such as initiatives to enhance awareness of symptoms such as PSD, as well as the development of specific guidelines on non-sensorimotor behavioural testing in the context of stroke, are needed to begin to address these devastating consequences of stroke.

## Supplemental Material

sj-pdf-1-jcb-10.1177_0271678X251317369 - Supplemental material for Behavioural assessment of neuropsychiatric outcomes in rodent stroke models Supplemental material, sj-pdf-1-jcb-10.1177_0271678X251317369 for Behavioural assessment of neuropsychiatric outcomes in rodent stroke models by Robert M Callaghan, Huiyuan Yang, Rachel D Moloney and Christian Waeber in Journal of Cerebral Blood Flow & Metabolism
