# Supplementary material for: Behavioral assessment of neuropsychiatric outcomes in rodent stroke models
Source: J Cereb Blood Flow Metab. 2025 Mar 20;45(7):1232–48. doi: 10.1177/0271678X251317369 (PMC11926818; doi:10.1177/0271678X251317369)
Supplement: sj-pdf-1-jcb-10.1177_0271678X251317369 - Supplemental material for Behavioural assessment of neuropsychiatric outcomes in rodent stroke models [file sj-pdf-1-jcb-10.1177_0271678X251317369.pdf]

Table 1 - Descriptions of non-sensorimotor behavioural tests reported in the publications analysed in the systematised review of the behavioural assessment of neuropsychiatric outcomes in rodent stroke models

Test	Description & Useful References	Apparatus
Morris water maze	Used to assess spatial learning and memory. The rodent is placed in a pool of water and required to find a hidden platform beneath the water in a predetermined time. Visual cues are placed around the pool and used by the rodent to navigate to the hidden platform. Cognitive flexibility can also be assessed by continually re-locating the hidden platform in the pool [1,2].	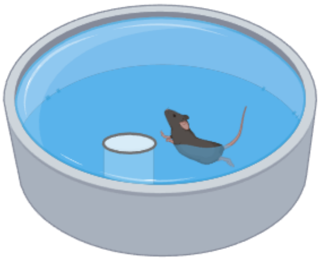
Y-maze	Used to assess short-term memory. The maze consists of three identical arms arranged in a Y shape. Working spatial memory can be assessed by allowing the rodent to freely explore the maze. Rodents with intact memory will show preference for less recently visited arms. Recognition memory can also be assessed by blocking one of the three arms in an initial trial, and reopened in a subsequent trial [3,4].	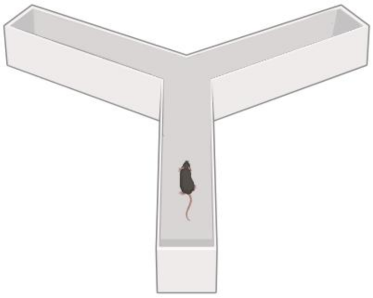
Barnes maze	Used to assess spatial learning and memory. The rodent is placed on a brightly lit open table with circular holes on its periphery. Connected to one of these holes is an "escape box" allowing shelter for the rodent from the aversive environment of the maze. Visual cues are employed to allow the rodent to navigate to this box. Memory is assessed based on the rodent's ability to find the escape box [5,6].	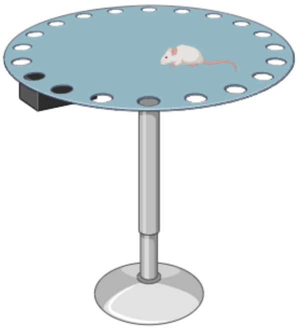
T-maze	Used to assess spatial learning and memory. Rodents are placed at the base of the maze and given the choice to enter a left or right arm. Working memory can be assessed through observing spatial alteration, while rewards placed in an arm can be used to evaluate reward-based learning [7,8].	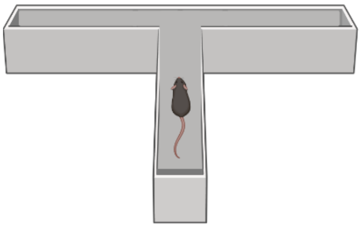

Radial arms maze	Used to assess spatial learning and memory. Rodents are placed in a central circular platform with a number of arms radiating out - each ending with a hidden food site. Working memory is assessed based on a rodent's ability to avoid re-entering already visited arms, while reference memory is assessed based on its ability to enter only food-containing arms [9,10].	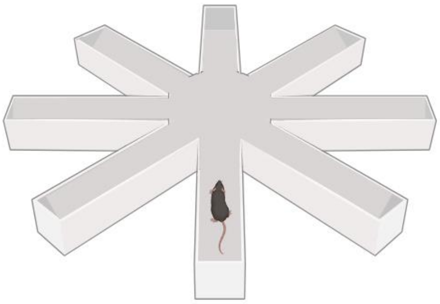
Lashley III maze	Used to assess spatial learning and memory. Rodents are placed in a maze containing four segements, and a goal box at the end. This test relies on the rodent's ability to learn the route to escape into a goal box through repeated trials. Alterations include constructing a pseudo-home cage at the end of the goal box to provide motivation for the rodent to escape the maze [11,12].	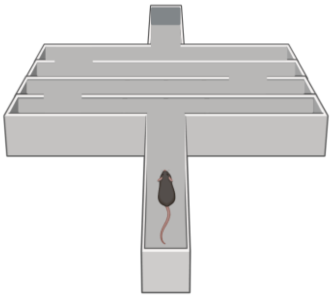
Novel object recognition	Used to assess spatial and recognition memory. Involves placing the rodent in an arena with two identical objects. Following an inter-trial period rodents re-enter the arena with one identical object replaced with a novel one. Memory is assessed based on the rodent's preference for the novel object. Short inter trial times (e.g. 1 hour) assesses short term memory while long times (e.g. 24 hours) assesses long-term memory [13,14].	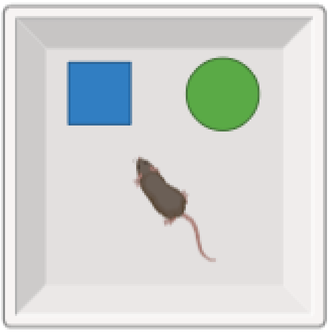
Novel odour recognition	Used to assess recognition memory. Involves placing the rodent in an arena with a familiar odour. Following an inter-trial period rodents are reintroduced to the apparatus along with both a familiar and novel odour. Memory is assessed based on the rodent's preference to explore the novel odour over the familiar odour. There are several variants of this paradigm including the spatial odour memory test, temporal odour memory test, and a "what-when-where" memory test [19,20].	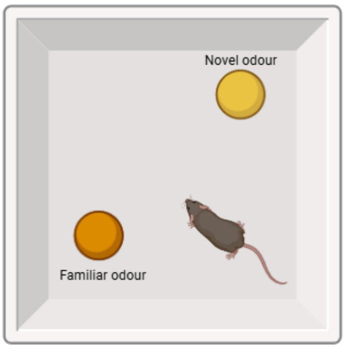

Novel location recognition	Used to assess spatial memory. Rodent is acclimatised to an arena containing two identical objects in relation to spatial environmental cues. During the inter-trial period the rodent is removed and one of the objects is moved to a different location within the arena. When the rodent is reintroduced spatial memory is assessed based on their preference to investigate the object in the novel location [24,25].	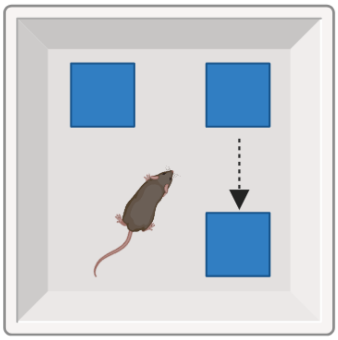
Passive avoidance test	Used to assess contextual fear memory. Involves putting rodent in a chamber consisting of two connected chambers, one brightly lit and another dark. Rodents will receive a shock upon entering the dark chamber and must learn to avoid entering this chamber [15,16].	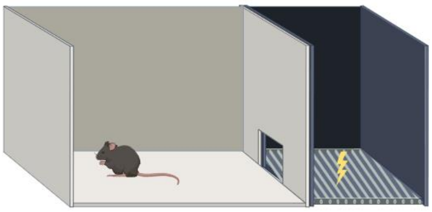
Fear conditioning test	Used to assess contextual fear memory. Involves putting rodent in a novel environment and providing an aversive stimulus such as a shock or sound. Upon re-entry into the novel environment rodents will elicit a freezing response -which is measured as an indicator of memory. Alterations to this test include the cued fear conditioning test, and delay and trace conditioning [17,18]	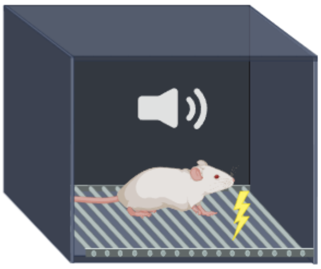
Touchscreen test	Used to assess a variety of cognitive domains using a touchscreen apparatus not unlike humans. Motivation for rodents is appetitive opposed to aversive. Learning and memory is assessed via visuomotor conditioning learning, autoshaping, and visual discrimination assays. Executive function and working memory can also be assessed through other assays [21,22].	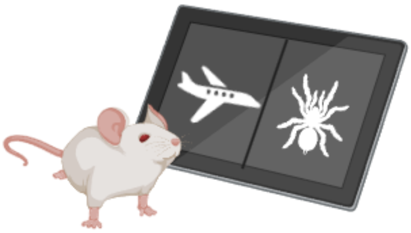

Three chamber test	Used to assess memory and sociability. Involves placing the rodent in an apparatus with three chambers. In the trial the rodent is accompanied in the chamber by a familiar and novel rodent. Sociability and memory is assessed on the rodent's preference to interacting with the novel rodent [12,23].	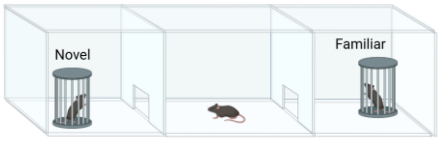
Open field test	Used to assess anxiety and locomotion. Involves placing a rodent in an open square or circular arena. Anxiety is assessed through outcome measures like time spent in the centre of the arena, circling behaviour, and number of faecal pellets. Locomotion is measured through walk speed, total distance travelled, and time spent mobile [26,27].	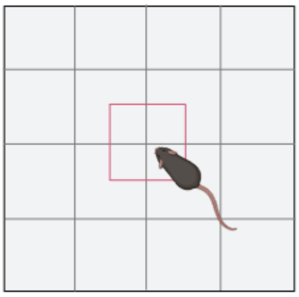
Elevated plus maze	Used to assess anxiety. Involves placing a rodent in a plus shaped maze that is elevated off the ground. This maze has two open arms and two closed arms. Rodents have an aversion towards open spaces and thus will naturally gravitate towards the less aversive enclosed arms. Anxiety is measured based on the time spent in the open versus closed arms of the elevated plus maze [8,28].	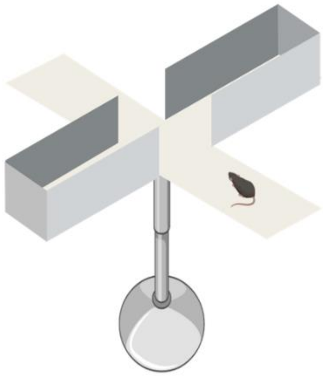
Sucrose preference test	Used to assess a phenotype of depression, particularly anhedonia. Mice are presented with two water bottles alongside one another. One bottle contains water, while the other contain a sucrose solution. Hedonic behaviour is expressed by a preference for the sucrose solution, which is measured by the ratio of sucrose solution consumed: total consumption (i.e., sucrose solution & water) [29,30].	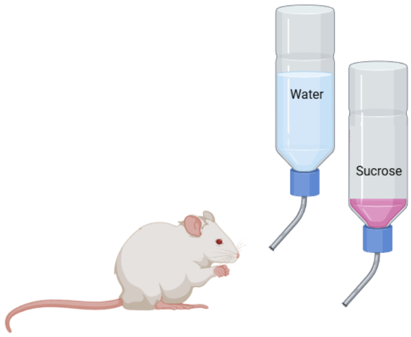

Forced swim test	Used to assess a phenotype of depression, particularly to measure the efficacy of antidepressants. Rodents are placed in a cylinder filled with water which they cannot escape. It is thought that immobility in this test represents a depressive phenotype, while swimming represents resilience to low mood [31,32].	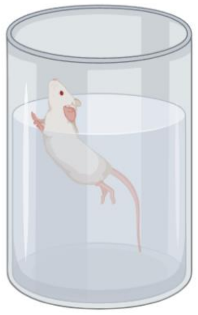
Tail suspension test	Used to assess a phenotype of depression, particularly to measure the efficacy of antidepressants. Mice are hung by their tail by a tube, elevated from the ground. Immobility represents a depressive phenotype while attempts to escape are considered to represent resilience to low mood. Rats should not be used for this test, as their heavier weight would put excessive strain on the tail causing pain and distress [33,34].	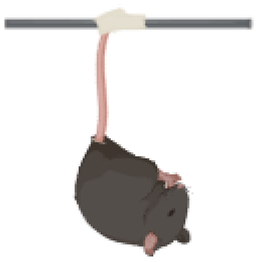

Table 2 – The scoring method used for individual non-sensorimotor behavioural tests reported in the publications analysed in the systematised review. Methods included the use of automated scoring software, and manual scoring by experimenters

Test	Automated	Manual	Not specified
Morris Water Maze	36	5	24
Y-Maze	3	5	11
Barnes Maze	1	1	5
T-Maze	0	0	4
Radial arms maze	0	0	1
Lashley III Maze	1	0	0
Novel object recognition	8	2	6
Passive avoidance test	4	0	6
Fear conditioning test	4	2	2
Novel odour recognition	0	0	2
Touchscreen test	2	0	0
Three chamber test	1	0	0
Novel location recognition	1	0	0
Open field test	32	5	11
Elevated plus maze	1	0	4
Sucrose preference test	0	2	0
Forced swim test	0	2	0
Tail suspension test	0	0	1
**Total**	**94**	**24**	**77**

Table 3 – Details of Inclusion/Exclusion Criteria reported in the publications analysed in the systematised review of the behavioural assessment of neuropsychiatric outcomes in rodent stroke models

Inclusion/Exclusion Criteria	No. of Papers
Successful ischaemic stroke model (i.e. predefined blood flow reductions, infarcts in areas outside region of interest, presence of frontal/hindlimb paresis post-stroke, absence of brain haemorrhage)	69
Behavioural test non-learners	17
Premature death	16
Humane endpoints	15
Excessive sensorimotor impairment	3
Requirement of paired data	2
Data point outliers	2
Technical issues specific to individual experiments (osmotic pump failure, poor electrocorticogram signal, excessive MRI noise, weight-based exclusion, transplant failure, failed transgene expression)	8

References

1. *Morris R. Developments of a water-maze procedure for studying spatial learning in the rat.* J Neurosci Methods *1984; 11: 47–60.*

2. *Schmidt A, Diederich K, Strecker J-K, et al. Progressive cognitive deficits in a mouse model of recurrent photothrombotic stroke.* Stroke *2015; 46: 1127–1131.*

3. *Kraeuter A-K, Guest PC, Sarnyai Z. The Y-Maze for Assessment of Spatial Working and Reference Memory in Mice. In: Guest PC (ed)* Pre-Clinical Models: Techniques and Protocols. *New York, NY: Springer, pp. 105–111.*

4. *Li F, Zhang Y, Li R, et al. Neuronal Serpina3n is an endogenous protector against blood brain barrier damage following cerebral ischemic stroke.* Journal of Cerebral Blood Flow & Metabolism *2022; 43: 241.*

5. *Barnes CA. Memory deficits associated with senescence: a neurophysiological and behavioral study in the rat.* J Comp Physiol Psychol *1979; 93: 74–104.*

6. *Sun H-S, Doucette TA, Liu Y, et al. Effectiveness of PSD95 inhibitors in permanent and transient focal ischemia in the rat.* Stroke *2008; 39: 2544–2553.*

7. *Deacon RMJ, Rawlins JNP. T-maze alternation in the rodent.* Nat Protoc *2006; 1: 7–12.*

8. *Prakash R, Li W, Qu Z, et al. Vascularization Pattern after Ischemic Stroke is Different in Control versus Diabetic Rats: Relevance to Stroke Recovery.* Stroke; a journal of cerebral circulation *2013; 44: 10.1161/STROKEAHA.113.001660.*

9. *Olton DS, Samuelson RJ. Remembrance of places passed: Spatial memory in rats.* Journal of Experimental Psychology: Animal Behavior Processes *1976; 2: 97–116.*

10. *Popa-Wagner A, Stöcker K, Balseanu AT, et al. Effects of granulocyte-colony stimulating factor after stroke in aged rats.* Stroke *2010; 41: 1027–1031.*

11. *Lashley KS, Ball J. Spinal conduction and kinesthetic sensitivity in the maze habit.* Journal of Comparative Psychology *1929; 9: 71–105.*

12. *Hosseini L, Karimipour M, Seyedaghamiri F, et al. Intranasal administration of mitochondria alleviated cognitive impairments and mitochondrial dysfunction in the photothrombotic model of mPFC stroke in mice.* Journal of Stroke and Cerebrovascular Diseases *2022; 31: 106801.*

13. *Ennaceur A, Delacour J. A new one-trial test for neurobiological studies of memory in rats. 1: Behavioral data.* Behavioural Brain Research *1988; 31: 47–59.*

14. *Huo K, Wei M, Zhang M, et al. Reduction of neuroinflammation alleviated mouse post bone fracture and stroke memory dysfunction.* J Cereb Blood Flow Metab *2021; 41: 2162–2173.*

15. *Ogren S, Stiedl O. Passive avoidance. In:* Encyclopedia of Psychopharmacology. *2015, pp. 1220–1227.*

16. *Zhuo Z, Wang H, Zhang S, et al. Selenium supplementation provides potent neuroprotection following cerebral ischemia in mice.* J Cereb Blood Flow Metab *2023; 43: 1060–1076.*

17. *Shoji H, Takao K, Hattori S, et al. Contextual and Cued Fear Conditioning Test Using a Video Analyzing System in Mice.* Journal of Visualized Experiments (JoVE) *2014; e50871.*

18. Torres-López C, Cuartero MI, García-Culebras A, et al. Ipsilesional Hippocampal GABA Is Elevated and Correlates With Cognitive Impairment and Maladaptive Neurogenesis After Cortical Stroke in Mice. Stroke *2023; 54: 2652–2665.*

19. Aqrabawi AJ, Kim JC. Behavioral Evaluation of Odor Memory in Mice. Bio Protoc *2018; 8: e3023.*

20. Zhang L, Chopp M, Zhang Y, et al. Diabetes Mellitus Impairs Cognitive Function in Middle-Aged Rats and Neurological Recovery in Middle-Aged Rats After Stroke. Stroke *2016; 47: 2112–2118.*

21. Horner AE, Heath CJ, Hvoslef-Eide M, et al. The touchscreen operant platform for testing learning and memory in rats and mice. Nat Protoc *2013; 8: 1961–1984.*

22. Sanchez-Bezanilla S, Hood RJ, Collins-Praino LE, et al. More than motor impairment: A spatiotemporal analysis of cognitive impairment and associated neuropathological changes following cortical photothrombotic stroke. J Cereb Blood Flow Metab *2021; 41: 2439–2455.*

23. Crawley JN. Designing mouse behavioral tasks relevant to autistic-like behaviors. Ment Retard Dev Disabil Res Rev *2004; 10: 248–258.*

24. Denninger JK, Smith BM, Kirby ED. Novel Object Recognition and Object Location Behavioral Testing in Mice on a Budget. Journal of Visualized Experiments (JoVE) *2018; e58593.*

25. Ahn SM, Jung DH, Lee HJ, et al. Contralesional Application of Transcranial Direct Current Stimulation on Functional Improvement in Ischemic Stroke Mice. Stroke *2020; 51: 2208–2218.*

26. Hall C, Ballachey EL. A study of the rat's behavior in a field. A contribution to method in comparative psychology. University of California Publications in Psychology *1932; 6: 1–12.*

27. Wang Y-C, Dzyubenko E, Sanchez-Mendoza EH, et al. Postacute Delivery of GABAA α5 Antagonist Promotes Postischemic Neurological Recovery and Peri-infarct Brain Remodeling. Stroke *2018; 49: 2495–2503.*

28. Handley SL, Mithani S. Effects of alpha-adrenoceptor agonists and antagonists in a maze-exploration model of 'fear'-motivated behaviour. Naunyn-Schmiedeberg's Arch Pharmacol *1984; 327: 1–5.*

29. Willner P, Towell A, Sampson D, et al. Reduction of sucrose preference by chronic unpredictable mild stress, and its restoration by a tricyclic antidepressant. Psychopharmacology *1987; 93: 358–364.*

30. Balkaya M, Kim I-D, Shakil F, et al. CD36 deficiency reduces chronic BBB dysfunction and scar formation and improves activity, hedonic and memory deficits in ischemic stroke. J Cereb Blood Flow Metab *2021; 41: 486–501.*

31. Porsolt RD, Le Pichon M, Jalfre M. Depression: a new animal model sensitive to antidepressant treatments. Nature *1977; 266: 730–732.*

32. Luo L, Li C, Deng Y, et al. High-Intensity Interval Training on Neuroplasticity, Balance between Brain-Derived Neurotrophic Factor and Precursor Brain-Derived Neurotrophic Factor in Poststroke Depression Rats. Journal of Stroke and Cerebrovascular Diseases *2019; 28: 672–682.*

33. Steru L, Chermat R, Thierry B, et al. The tail suspension test: A new method for screening antidepressants in mice. Psychopharmacology *1985; 85: 367–370.*

34. Chokkalla AK, Jeong S, Mehta SL, et al. *Cerebroprotective Role of N6-Methyladenosine Demethylase FTO (Fat Mass and Obesity-Associated Protein) After Experimental Stroke.* Stroke *2023; 54: 245–254.*

Acknowledgements

Morris Water Maze Figure Created in BioRender. Callaghan, R. Moloney, R. Waeber, C. (2024) https://BioRender.com/n42y547

Y Maze Figure Created in BioRender. Callaghan, R. Moloney, R. Waeber, C. (2024) https://BioRender.com/x55d439

Barnes Maze Figure Created in BioRender. Callaghan, R. Moloney, R. Waeber, C. (2024) https://BioRender.com/o18q294

T Maze Figure Created in BioRender. Callaghan, R. Moloney, R. Waeber, C. (2024) https://BioRender.com/a36h031

Radial Arms Maze Figure Created in BioRender. Callaghan, R. Moloney, R. Waeber, C. (2024) https://BioRender.com/i50x835

Lashley III Maze Created in BioRender. Callaghan, R. Moloney, R. Waeber, C. (2024) https://BioRender.com/j48c338

Novel Object Recognition Figure Created in BioRender. Callaghan, R. Moloney, R. Waeber, C. (2024) https://BioRender.com/g29n316

Novel Odour Recognition Figure Created in BioRender. Callaghan, R. Moloney, R. Waeber, C. (2024) https://BioRender.com/x66k306

Novel Location Recognition Figure Created in BioRender. Callaghan, R. Moloney, R. Waeber, C. (2024) https://BioRender.com/w55p392

Passive Avoidance Test Figure Created in BioRender. Callaghan, R. Moloney, R. Waeber, C. (2024) https://BioRender.com/i30t557

Fear Conditioning Test Figure Created in BioRender. Callaghan, R. Moloney, R. Waeber, C. (2024) https://BioRender.com/m70f082

Touchscreen Test Figure Created in BioRender. Callaghan, R. Moloney, R. Waeber, C. (2024) https://BioRender.com/a66j287

Three Chamber Test Figure Created in BioRender. Callaghan, R. Moloney, R. Waeber, C. (2024) https://BioRender.com/p34g363

Open Field Test Figure Created in BioRender. Callaghan, R. Moloney, R. Waeber, C. (2024) https://BioRender.com/n49b434

Elevated Plus Maze Created in BioRender. Callaghan, R. Moloney, R. Waeber, C. (2024) https://BioRender.com/q06j429

Sucrose Preference Test Figure Created in BioRender. Callaghan, R. Moloney, R. Waeber, C. (2024) https://BioRender.com/o72z433

Forced Swim Test Figure Created in BioRender. Callaghan, R. Moloney, R. Waeber, C. (2024) https://BioRender.com/t08m334

Tail Suspension Test Figure Created in BioRender. Callaghan, R. Moloney, R. Waeber, C. (2024) https://BioRender.com/x94u992
